# Quantitative relationship between grip strength and quality of life in the older adult based on a restricted cubic spline model

**DOI:** 10.3389/fpubh.2024.1417660

**Published:** 2024-09-17

**Authors:** Fanhao Meng, Yunqing Zhang, Chaoxing Liu, Cailiang Zhou

**Affiliations:** ^1^School of Strength and Conditioning, Beijing Sport University, Beijing, China; ^2^China Basketball College, Beijing Sport University, Beijing, China; ^3^School of Leisure and Tourism, Beijing Sport University, Beijing, China; ^4^School of Sports Science, Beijing Sport University, Beijing, China

**Keywords:** quality of life, SF-36, muscle strength, restricted cubic spline model, older adult

## Abstract

**Background:**

Grip strength have been showed diverse associations with quality of life for the older adult population in the literature, still there is lack of evidence of the threshold value of grip strength for maintaining good quality of life in older adults. The purpose of this study was to study the dose-effect relationship between grip strength and quality of life in the older adult, and to explore the factors affecting quality of life in the older adult, so as to provide effective theoretical basis for realizing healthy aging.

**Methods:**

A total of 105 older adult people over 60 years old were selected from 3 communities in Beijing. Grip strength was measured by hand dynamometer apparatus and quality of life was assessed by 36-item Short-Form (SF-36). On the basis of controlling confounding factors, the dose-effect relationship between grip strength and quality of life was analyzed with the restricted cubic spline model.

**Results:**

The results showed that there was a dose-effect relationship between grip strength and physical component summary (PCS) (*p* < 0.01). However, grip strength was not significantly associated with mental component summary (MCS) (*p* > 0.05). The threshold value of grip strength for male and female is 34.75 and 23.2 kg, for normal weight group and overweight and obesity group is 24.82 and 29.00 kg, for 60–69, 70–79, 80+ years group is 24.88, 23.37, and 22.97 kg, respectively. When the grip strength value is lower than the threshold value, the increase of grip strength was related to significant improvement of quality of life of the older adult, and when the grip strength value is higher than the threshold value, the quality of life can be maintained in good condition.

**Conclusion:**

A dose-effect relationship was found between grip strength and physical health in quality of life. Results of our study indicated that the grip strength of the older adults needed to be greater than certain threshold values to maintain good quality of life.

## 1 Introduction

Quality of life is a multidimensional concept that has become increasingly important in recent years due to its role in people’s social and mental health (1). One of the important dimensions of quality of life is physical health, considering the growing population of the older adult and the fact that the health of society depends on their well-being, it is necessary to pay more attention to their quality of life (2, 3). Relevant studies have shown that strength is positively associated with quality of life in older adults (4–6). Muscle strength is a key factor in maintaining physical function and mobility in older adults (7). Loss of muscle mass and strength with age is a major cause of decline in physical functioning in older adults (8), which may adversely reflect on quality of life among older adults (9), and grip strength is an effective indicator to determine physical health, it is increasingly used as a detection of overall muscle strength and function, and it is associated with the aging of several body systems, which can be used as a simple and practical indicator to identify physical decline and quality of life in the older adult (10–12).

Exploring the intervenable factors affecting quality of life in older adults will provide an effective policy basis for achieving healthy aging. Previous studies have shown that grip strength in older adults is one of the important variables affecting quality of life, but the evidence on the strength of the association between the two is inconsistent and there is currently no consensus on a threshold value for grip strength in older adults (13). In conclusion, the strength of the association between grip strength and quality of life in older adults has been presented inconsistently in different studies, and the present study, based on obtaining data on grip strength and quality of life in older adults, controlling for major confounders and analyzing the quantitative relationships between them.

## 2 Objects and methods

### 2.1 Subjects

Older adults aged 60 years or older were selected from three communities in Beijing for this study. The inclusion and exclusion criteria for the subjects were age ≥ 60 years, those who had lived in the local community for more than 5 years and were willing to participate in this study, and those who were unable to perform the relevant physical activities within the last month due to illness or other reasons, respectively.

### 2.2 Methods of data collection

A questionnaire on basic personal information (sex, date of birth, marital status, education level, presence of chronic diseases, etc.) was designed by the researcher.

Participants’ weight and height were measured in light clothing without shoes. Body mass index (BMI) was calculated with the weight of the individual in kilograms divided by the square of the individual’s standing height in meters (kg/m^2^). Normal weight was defined as a BMI of 20.0–24.9 kg/m^2^, overweight as a BMI of 25.0–29.9 kg/m^2^, and obesity as a BMI of 30.0 kg/m^2^ or more (14). Chronic diseases were determined either by the self-reported response or confirmed diagnosis from a doctor, including diabetes, high blood pressure, osteoporosis, asthma, etc.

Grip strength was measured by a Jamar^®^ hydraulic hand dynamometer apparatus (15), in which subjects’ feet are naturally separated into an upright position with their arms drooping. Hold the grip gauge tightly with one hand and calculate the scale of the grip gauge pointer. Each subject was tested 3 times, and the maximum value of the most powerful hand was taken from the subject, and the value of grip strength was scored in the way of standard percentage, and the scoring range was ($\bar{x}$ ± 3 s) (16).

Quality of life was measured using the SF-36 scale (The Medical Outcome Study 36-item short form health survey). The SF-36 scale consists of 8 dimensions: physical function (PF), role physical (RP), bodily pain (BP), general health (GH), vitality (V), social functioning (SF), role emotional (RE), and mental health (MH). It comprises a total of 35 entries, including a self-reported health change entry (17). The SF-36 scale has been found to have good reliability and validity (18). The eight dimensions are grouped into two categories as component measures: the physical component summary (PCS) and the mental component summary (MCS). In this study, the two component measures were used as the primary indicators to evaluate the quality of life. The formulas for the two component measures are as follows: PCS = (standardized score for each dimension × physical factor score corresponding to each dimension) × 10 + 50 and MCS = (standardized score for each dimension × mental factor score corresponding to each dimension) × 10 + 50. Reliability analyses of the dimensions of the SF-36 showed that the Cronbach coefficients for the eight dimensions of PF, RP, BP, GH, V, SF, RE, and MH were 0.83, 0.92, 0.85, 0.69, 0.70, 0.83, 0.88, and 0.68 respectively, with values of greater than or close to 0.70. The structural validity evaluations using factor analysis showed that the common factors generated by factor analysis were generally consistent with the theoretical structure. The structural validity evaluation using factor analysis showed that the common factors generated by factor analysis were basically consistent with the theoretical structure.

### 2.3 Quality control

The purpose, significance and methodology of the survey were clarified to the respondents before the survey and test so that they could fully understand them in order to seek their cooperation. During the on-site survey and test, the surveyor carefully explained the survey scale and test notes to the respondents. The survey is conducted face-to-face, and the investigator fills out the questionnaire based on the respondents’ answers. As required by local legislation and institutions, our study does not require further ethics committee approval as it does not involve clinical trials and is not unethical. All participants provided informed consent prior to participating in the study. The anonymity and confidentiality of participants are guaranteed and participation is entirely voluntary.

### 2.4 Methods of statistical analysis

Epidata 3.0 software (The Epidata Association, Odense, Denmark) was used to create the database, and R 4.2.3 software (R CoreTeam, Vienna, Austria) was used for statistical analysis. Information on numerical variables was expressed as $\bar{x}\pm s$ and information on categorical variables was expressed as frequencies. Comparisons of two population means were performed using the t-test, and comparison between groups of categorical data were performed using the Chi-squared test. The relationships between grip strength and quality of life for the sub-groups were analyzed using restricted cubic spline models and conducted with 4 knots at the 5th, 35th, 65th, and 95th centiles to flexibly model the association, controlling for confounders.

## 3 Results

### 3.1 Sociological characteristics of the population studied

Of the 105 valid samples, 42 (40%) were male and 63 (60%) were female. The youngest was 60 years old and the oldest was 88 years old, with a median age of 70 years and an interquartile spacing of 15 years. According to the age group, there were 54(51%) persons aged 60–69 years old, 29 persons aged 70–79(51%) years old and 22(21%) persons aged 80 years old or above. The educational level was 57 (54%) in high school and below, and 48 (46%) in college and above. 89 (85%) were married, 16 (15%) were divorced or widowed. Among the subjects, 48(46%) were normal weight, 57 (54%) were overweight and obesity. Of all the study subjects, 74(70%) had chronic diseases and 31(30%) had no chronic diseases. There was a significant difference between male and female participants in terms of age, height, weight, education level, marital status, and chronic diseases (*p* < 0.05). There was no statistically significant difference in body mass index, and as expected, grip strength was significantly higher in male participants than in female participants (*p* < 0.01). The characteristics of the study population are summarized in Table 1.

**Table 1:** Summary characteristics of study participants.

	Males (*N* = 42)	Females (*N* = 63)	*p*-value
Age (years) [mean (SD)]	73.40 ± 8.08	70.24 ± 7.26	0.044*
Height (cm) [mean (SD)]	169.97 ± 6.47	158.25 ± 5.88	<0.001**
Weight (kg) [mean (SD)]	71.45 ± 10.02	60.50 ± 10.05	<0.001**
Body Mass Index (kg/m^2^) [mean (SD)]	24.67 ± 2.66	24.00 ± 3.30	0.255
Grip strength (kg) [mean (SD)]	34.60 ± 8.64	23.69 ± 5.62	<0.001**
Educational level			0.011*
High school and below [n (%)]	16 (38.1%)	41 (65.1%)	
College and above [n (%)]	26 (61.9%)	22 (34.9%)	
Marriage statues			0.015*
Married [n (%)]	40 (95.2%)	49 (77.8%)	
Divorced or widowed [n (%)]	2 (4.8%)	14 (22.2%)	
Chronic disease statues			0.045*
Yes [n (%)]	25 (59.5%)	49 (77.8%)	
No [n (%)]	17 (40.5%)	14 (22.2%)	

*P < 0.05, **p < 0.01.

### 3.2 Quality of life of older adults

The results of the eight dimensions of quality of life and the two component scores for older adults are presented in Table 2. Men scored higher in the dimensions of bodily pain, vitality, and emotional role compared to women (*p* < 0.05), but no statistically significant differences were observed between men and women in the other dimensions and two component summary scores (*p* > 0.05).

**Table 2:** Quality of life of older adults [mean (SD)].

Variable name	All (*n* = 105)	Male (*N* = 42)	Female (*N* = 63)	*t*	*P*
Physical function	82.71 ± 17.51	85.36 ± 16.21	80.95 ± 18.25	1.27	0.208
Role physical	75.95 ± 39.06	83.33 ± 33.89	71.03 ± 41.69	1.59	0.114
Bodily pain	73.09 ± 24.17	79.45 ± 23.06	68.84 ± 24.14	2.25	0.027*
General health	58.16 ± 20.20	61.67 ± 18.55	55.83 ± 21.05	1.46	0.147
Vitality	77.86 ± 19.89	83.69 ± 16.01	73.97 ± 21.35	2.52	0.013*
Social functioning	75.13 ± 20.36	75.93 ± 20.37	74.60 ± 20.49	0.32	0.746
Role emotional	80.32 ± 35.71	88.89 ± 29.14	74.60 ± 38.67	2.04	0.044*
Mental health	82.40 ± 17.06	85.14 ± 16.91	80.57 ± 17.04	1.35	0.180
PCS	49.74 ± 10.22	51.65 ± 9.26	48.46 ± 10.69	1.58	0.117
MCS	50.39 ± 10.13	52.54 ± 8.46	48.95 ± 10.93	1.80	0.076

*p < 0.05.

### 3.3 Relationship between grip strength and quality of life in the older adult

The results of the analysis using the restricted cubic spline model and adjusting for the confounders of marital status, educational level and chronic disease status, suggested a non-linear(L-shaped) association of grip strength with PCS in different sex, body mass index and age group. There was no quantitative relationship between grip strength and the MCS in older adults (*p* > 0.05).

#### 3.3.1 Relationship between grip strength and quality of life in different sex groups

**Figure 1:**
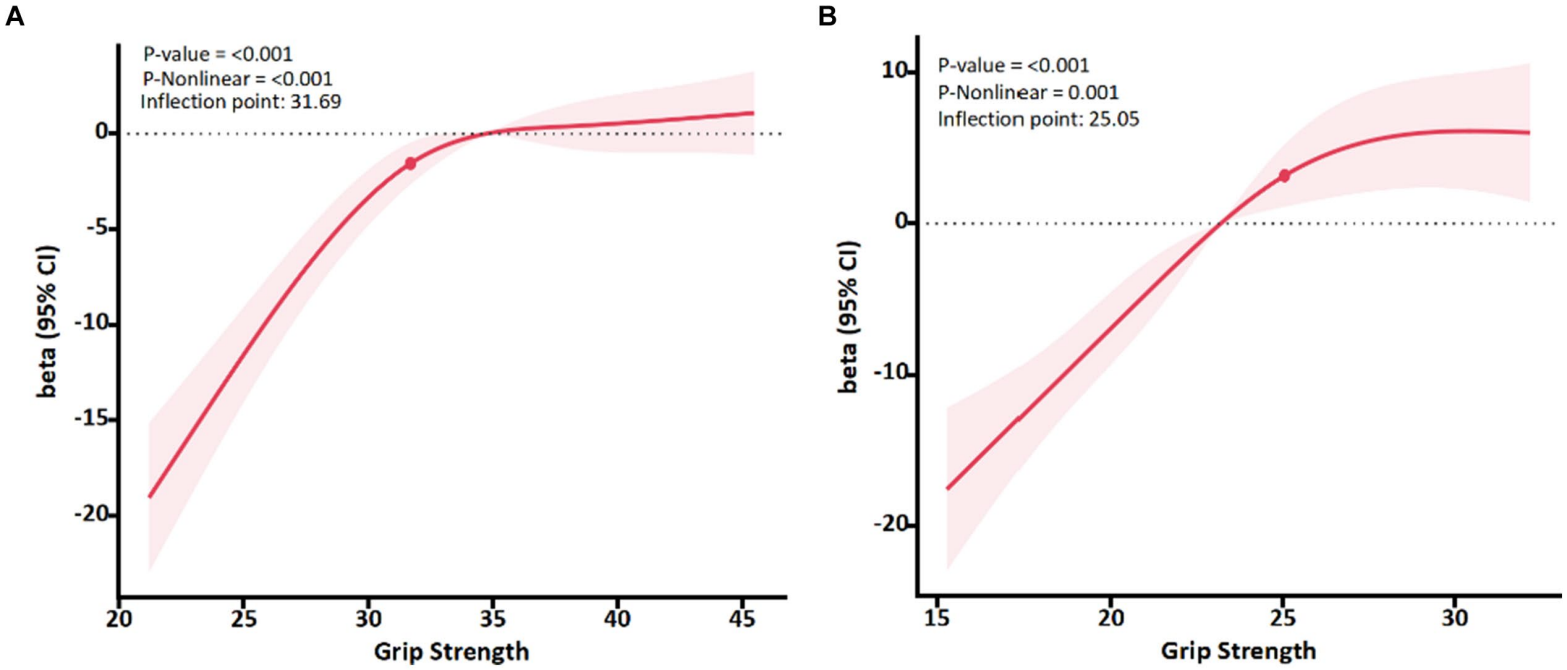
Association between Grip strength and PCS with the RCS function in male and female. Linear regression model was adjusted for age, body mass index, marital status, educational level and chronic disease status. Results with 95% Confidence interval.

Results of the dose-effect analysis showed that in both men and women, there was a dose-effect relationship between grip strength and PCS (*p* < 0.01) (Figure 1). When the grip strength of men and women is lower than 31.69 kg and 25.05 kg, with the increase of grip strength level, the PCS of the older adult improves rapidly. When the grip strength was greater than 31.69 kg and 25.05 kg, the PCS slowed down.

#### 3.3.2 Relationship between grip strength and quality of life in different body mass index groups

**Figure 2:**
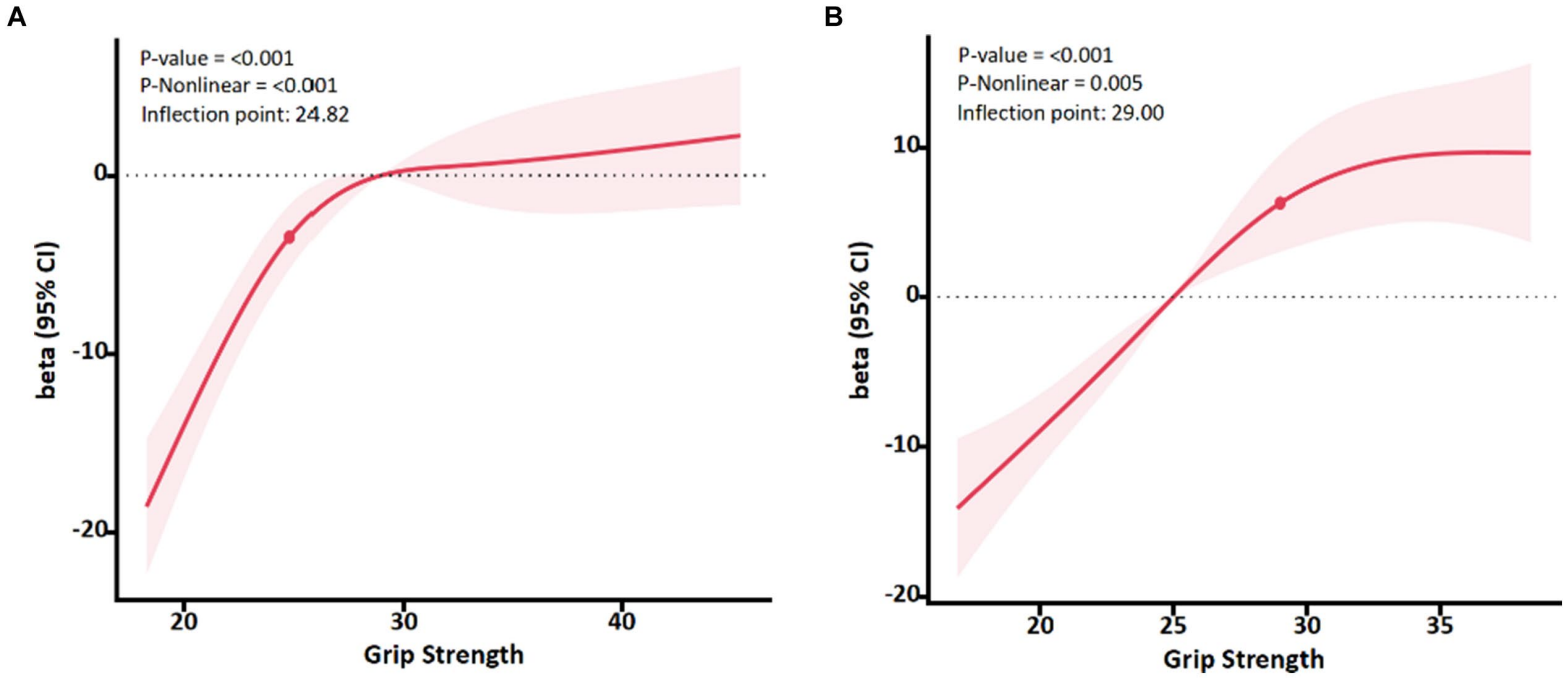
Association between Grip strength and PCS with the RCS function in normal weight and overweight group. Linear regression model was adjusted for age, sex, marital status, educational level and chronic disease status. Results with 95% Confidence interval.

In this study, only two older adults had a BMI over 30.0 kg/m^2^, so overweight and obesity were included in one group. Results of the dose-effect analysis showed that in two groups, there was a dose-effect relationship between grip strength and PCS (*p* < 0.01) (Figure 2). When the grip strength of normal weight and overweight group is lower than 24.82 and 29.00 kg, with the increase of grip strength level, the PCS of the older adult improves rapidly. When the grip strength was greater than the inflection point, the PCS slowed down.

#### 3.3.3 Relationship between grip strength and quality of life in different age groups

**Figure 3:**
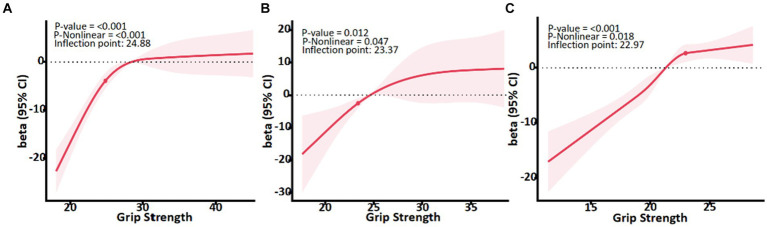
Association between Grip strength and PCS with the RCS function in male and female. Linear regression model was adjusted for sex, body mass index, marital status, educational level and chronic disease status. Results with 95% Confidence interval.

Results of the dose-effect analysis showed that in three age groups, there was also a dose-effect relationship between grip strength and PCS (*p* < 0.05) (Figure 3). When the grip strength of 60–69, 70–79, and over 80 years group is lower than 24.88, 23.37, and 22.97 kg, respectively. With the increase of grip strength level, the PCS of the older adult improves rapidly. When the grip strength was greater than the inflection point, the PCS slowed down.

## 4 Discussion

In this study, grip strength was chosen as the main strength measure because it is clearly related to mobility in the older adult and is easy to use in clinical and community settings (19). A recent narrative review examining the relationship between grip strength and health status in older adults concluded that g strength, as a biomarker, largely explains and predicts overall strength, upper limb function, bone mineral density, fractures, falls, malnutrition, cognitive impairment, depression, sleep problems, diabetes, and quality of life in older adults (13). And another study (20) also found that there is a positive association between grip strength and quality of life in the older adult (OR per kilogram decrease in grip strength = 1.13, 95% CI = 1.06–1.19), which also means that people with weaker muscle strength have a greater impact on quality of life, which may be due to weak grip strength and inability to carry out activities of daily living. Among Chinese community-based older adult, Xie and Ma (21) studied 400 older adult people over 80 years old from Shanghai, China, and the study also found that the stronger the grip strength, the better the overall quality of life (*β* = 4.40, *p* < 0.001). There are also studies that show (20, 22), Men with lower grip strength were more likely to report poor overall GH in the SF-36 domain and lower physical function and general health perceptions scores. Similarly, Wu et al. (23) combined 42 studies through meta-analysis and found that for every 5 kg reduction in grip strength in the older adult, the risk of all-cause mortality and cardiovascular disease increased by 1.16 and 1.21 times, respectively, and also believed that grip strength has been linked to risk of adverse health outcomes. Women’s findings were similar to men’s, but women with weaker grip strength were more likely to have lower physical function, vitality, and body pain scores in the SF-36 domain, as well as decreased grip strength, which has been linked to aging in multiple body systems. In this study, the average scores of body pain, vitality and role emotional in men were higher than those in women, and the average grip strength in men was higher than that in women, which was similar to the results of previous studies. In the process of aging, the change of physical function in the older adult is often caused by the decline of skeletal muscle mass and function and this in turn is related to the need for support services, long term care and reduced quality of life (24). This is because older adult people with weak hand grip strength may have difficulty in completing multiple activities in daily life, such as grocery shopping or housework, which are key parts of daily living activities, and limited daily living activities are associated with quality of life differences in older adult people (25). This shows that maintaining grip strength can improve the quality of life, enhancing the possibility of being productive in old age.

Grip strength is associated with various physical activities, and allow the use of norm data to understand sex, body mass index and age differences, as test results can differ significantly between these groups. In the evaluation of grip strength of different gender, Wang and Chen (26) determined the critical value of grip strength required by the older adult to handle heavy tasks (for example, lifting or carrying 11 kg of objects), which is 28.5 kg for men and 18.5 kg for women, which shows that grip strength can be used as a test indicator of upper limb strength. In the grip strength and older adult walking activities, although the grip strength is not directly required for functional activities such as walking, the grip strength can be distinguished according to the mobility of the older adult. Forrest et al. (27) research found that in the older adult Americans with limited physical activity, When they stand from chairs, walk, and climb steps poorly, it is often accompanied by a significant lack of grip strength. In some studies that identified slower walking speeds (< 0.80 m/s), grip strength thresholds ranged from 23.2 to 39.0 kg in men. Thresholds for women range from 15.9 to 22.0 kg (28–30). In the past, sarcopenia was defined by grip strength. The European Working Group on Sarcopenia in the older adult defined “weakness” based on a grip strength of less than 30 kg for men and less than 20 kg for women (31). Chun et al. (32) analyzed grip strength and strength divided by weight in 1,273 men and 1,436 women to explore the relationship between sarcopenia and low quality of life. The study showed that these two indicators showed similar correlation with quality of life in both sexes, and it was also believed that poor grip strength to restricted activities of daily living were the main reasons for low quality of life in the older adult. This shows that exploring the critical value of grip strength in the older adult population will help improve the physical activity and health status of the older adult, thereby promoting their quality of life. Identifying specific grip strength thresholds to identify which older adults have weak grip strength that may lead to limited walking, such inflection points may help identify which populations may benefit from interventions to improve muscle strength and function. This is also similar to the inflection points between men and women in this study. We believes that before the grip strength of the older adult is 31.69 and 25.05 kg respectively, the PCS of the older adult will increase significantly, which means that as the grip strength increases, the older adult will rapidly reduce the risk of health status.

Obese people require more muscle strength to move their body mass than normal-weight people (33, 34), so in the older adult population, it may be necessary to examine the grip strength inflection points for the fastest quality of life improvement in normal-weight, overweight, and obese people separately. Based on a representative population-based study, the optimal grip strength inflection point s for the likelihood of limited activity in older men and women was identified. Grip strength inflection points increased with body mass index (35). In this study, body mass index was divided into normal and overweight groups, which also met the inflection point of grip strength increased with the increase of body mass index. When the grip strength of normal weight group and overweight group was lower than 24.82 and 29.00 kg, the PCS of older adult people improved rapidly with the increase of grip strength level. After age 60, grip strength decreases by an average of 5–6 kg for men and 3–4 kg for women every 10 years or so (13, 36). Reduced grip strength may accelerate dependence on activities of daily living, which can negatively impact quality of life (20, 37). In this study, maintaining the threshold of grip strength at 24.88, 23.37, and 22.97 kg in 60–69, 70–79, 80+ age groups can delay the decline of quality of life. And these values can also be used as a reference indicator for the physical health of the older adult. WHO’s “Global Physical Activity for Health Recommendations” states that “adults aged 65 and over should participate in 150 min of moderate-intensity or 75 min of vigorous-intensity aerobic activities per week, and two or more days of muscle strengthening activities.” (38). In order to prevent the quality of life of the older adult from decreasing with the reduction of grip strength, the older adult can increase or maintain muscle mass through resistance exercises. In addition to the impact of physical health, quality of life is also affected by environmental and social factors (39), Therefore, this study in the control of chronic diseases, marriage statues and other confounding factors to explore the impact of the older adult quality of life grip strength threshold value.

The results of our study showed that there is no correlation between the grip test and the mental components (MCS), which is consistent with the results of other study (11). However, some studies have explored the relationship between physical activity and subjective well-being of the older adult in low-and middle-income countries. Higher grip strength will increase the probability of happiness, and those who live with their families have better mental status. This may be attributed to in family activities, mental health and grip strength will be improved to a certain extent, and active participation in family and social activities may have a positive contribution to the mental health of the older adult (40). And good social network and social connection can improve the well-being and mental health of the older adult (10). However, this study did not find that the grip strength level of the older adult and the mental component summary is related.

The main limitation of this study is that the sample obtained cross-sectional data, so it is not possible to establish a causal relationship between grip strength and quality of life, while low quality of life may also contribute to inactivity and loss of muscle function. The results of this study showed that the grip strength and the quality of life of the older adult showed a strong dose-effect relationship, and the threshold of grip strength was relatively high compared with previous studies, which may be due to the overall high level of grip strength of the sample in this survey. The average grip strength of men and women was 31.69 and 25.05 kg, respectively, according to the results of 105 residents in 3 communities in Beijing. Therefore, the results of this study suggest that for the older adult population in urban communities with relatively high grip strength, there may be a strong dose-effect relationship between grip strength and quality of life. The results of the study need to be further demonstrated by cohort studies or experimental studies in different regions and people with grip strength levels. Addressing this in more detail requires cohort follow-up to obtain longitudinal data, which in turn examines the relationship between baseline grip strength and subsequent quality of life.

## 5 Conclusion

In conclusion, this study shows that there is a dose-effect relationship between the grip strength of the older adult and physical health and the grip strength can be used as an predictive index to evaluate the quality of life. To maintain a good quality of life, it is suggested that the grip strength of male and female older adult population need to be maintained at least at 31.69 and 25.05 kg. Grip strength should be maintained at a minimum of 24.82 kg for normal weight older adults and 29.00 kg for overweight and obesity older adults. Grip strength should be maintained at a minimum of 24.88, 23.37, and 22.97 kg in the 60–69, 70–79, and 80+ age groups, respectively.

## Data Availability

The original contributions presented in the study are included in the article/supplementary material, further inquiries can be directed to the corresponding author.
